# Evoked potentials after painful cutaneous electrical stimulation depict pain relief during a conditioned pain modulation

**DOI:** 10.1186/s12883-017-0946-7

**Published:** 2017-08-29

**Authors:** Oliver Höffken, Özüm S. Özgül, Elena K. Enax-Krumova, Martin Tegenthoff, Christoph Maier

**Affiliations:** 1 Department of Neurology, Berufsgenossenschaftliches Universitätsklinikum Bergmannsheil GmbH, Ruhr-University Bochum, Bürkle-de-la-Camp-Platz 1, 44789 Bochum, Germany; 2 Department of Pain Medicine, Berufsgenossenschaftliches Universitätsklinikum Bergmannsheil GmbH, Ruhr-University Bochum, Bürkle-de-la-Camp-Platz 1, 44789 Bochum, Germany

**Keywords:** CPM, Electrically evoked potentials, Cold pressor test, Pain relief

## Abstract

**Background:**

Conditioned pain modulation (CPM) evaluates the pain modulating effect of a noxious conditioning stimulus (CS) on another noxious test stimulus (TS), mostly based solely on subjective pain ratings. We used painful cutaneous electrical stimulation (PCES) to induce TS in a novel CPM-model. Additionally, to evaluate a more objective parameter, we recorded the corresponding changes of cortical evoked potentials (PCES-EP).

**Methods:**

We examined the CPM-effect in 17 healthy subjects in a randomized controlled cross-over design during immersion of the non-dominant hand into 10 °C or 24 °C cold water (CS). Using three custom-built concentric surface electrodes, electrical stimuli were applied on the dominant hand, inducing pain of 40–60 on NRS 0–100 (TS). At baseline, during and after CS we assessed the electrically induced pain intensity and electrically evoked potentials recorded over the central electrode (Cz).

**Results:**

Only in the 10 °C-condition, both pain (52.6 ± 4.4 (baseline) vs. 30.3 ± 12.5 (during CS)) and amplitudes of PCES-EP (42.1 ± 13.4 μV (baseline) vs. 28.7 ± 10.5 μV (during CS)) attenuated during CS and recovered there after (all *p* < 0.001). In the 10 °C-condition changes of subjective pain ratings during electrical stimulation and amplitudes of PCES-EP correlated significantly with each other (*r* = 0.5) and with CS pain intensity (*r* = 0.5).

**Conclusions:**

PCES-EPs are a quantitative measure of pain relief, as changes in the electrophysiological response are paralleled by a consistent decrease in subjective pain ratings. This novel CPM paradigm is a feasible method, which could help to evaluate the function of the endogenous pain modulation processes.

**Trial registration:**

German Clinical Trials Register DRKS-ID: DRKS00012779, retrospectively registered on 24 July 2017.

## Background

Conditioned pain modulation (CPM) describes the analgesic effect of a noxious conditioning stimulus (CS) on a noxious test stimulus (TS), based on the diffuse noxious inhibitory control [1–3]. Impaired endogenous pain inhibition has been demonstrated in several chronic pain states [4]. Furthermore, ineffective endogenous analgesia seems to represent a risk for developing chronic postoperative pain and opioid-induced hyperalgesia [5–8]. Remarkably, patients with painful diabetic neuropathy and insufficient endogenous analgesia respond better to duloxetine, a serotonin-noradrenalin-reuptake-inhibitor supposed to enhance the function of the descending inhibitory pathways [5].

Different fMRI studies focused on the neural mechanism of CPM. For example, using heat as TS and intramuscular injection of hypertonic saline as CS, the signal intensity increases during each TS in the presence of the CS were reduced in three brainstem regions: the caudalis subdivision of the spinal trigeminal nucleus, i.e., the primary synapse, the region of the subnucleus reticularis dorsalis and in the dorsolateral pons near the parabrachial nucleus [9]. These changes also correlated with the magnitude of analgesia. A further study using phasic heat as TS and heterotopic ice-cold as CS reported the psychophysical effect to be directly proportional to the cold-induced modulation of the laser-induced BOLD response in left posterior insula/SII, correlating also with changes in the anterior cingulate, orbitofrontal and lateral prefrontal cortices [10]. Obermann et al. combined functional magnetic resonance imaging and electrically evoked pain- related potentials (PREP) in healthy subjects showing that increasing stimulus intensity results in a similar increase of pain ratings, PREP, and BOLD responses within the pACC (posterior part of the anterior cingulate cortex), PCC (posterior cingulate cortex), insula, and somatosensory cortex (SII) [11].

Hitherto, there is no commonly accepted protocol for CPM-assessment [12]. Numerous paradigms with different TS and CS (pressure, heat, cold, ischemia, electricity, laser) have been introduced, with high variability in the magnitude of the CPM-effect across subjects and studies [13]. Several studies have evaluated the CPM-effect using electrophysiological findings to objectively quantify the CPM effect, based on the spinal nociceptive flexion reflex (changes of amplitude [14] or threshold [15]), the R2-response of the blink reflex [16], as well as on sensory evoked potentials (SEP) after CO_2_-laser stimulation [17–19], electrical tooth stimulation [20, 21], contact-heat stimulation [2] and chemonasal CO_2_-stimulation [22]. All of them showed significant effects across the study populations, however, they report of variable effects on individual subject level, and not all studies reported the number of subjects in whom the CPM-effect was detectable [23]. This is an important issue, because under optimal circumstances one needs a CPM protocol using reliable test and conditioning stimuli, which, as a control condition, is also able to elicit a CPM-effect in healthy subjects. Otherwise, in case that during CPM assessment a patient has no CPM-effect, one would not be able to differentiate whether this is due to methodological reasons or whether it is an abnormal finding due to the underlying disease.

Until now, and despite their clinical relevance in the assessment of small fiber function based on the pain-related evoked potentials [24–27], and the early scientific interest in electrically induced cortical potentials [28], the CPM-effect has not been investigated using painful cutaneous electrical stimulation (PCES) induced by K^2^ stimulation electrodes generating evoked potentials (PCES-EP) as TS. PCES-EP are recorded after activation of intraepidermal nociceptive fibers using custom-built concentric surface electrodes with small anode-cathode distance (K^2^ stimulation electrodes) [29], inducing a pinprick-like sensation. Although estimated mean conduction velocity was reported to be 11.61 ± 5.12 m/s, which is close to conduction velocity of A-delta fibers [29], this type of stimulation cannot exclude the possibility of low-threshold mechanoreceptors being involved. Interestingly, the individual pain ratings during PCES and amplitudes of PCES-EP have been shown to correlate significantly [29, 30]. Therefore, PCES-EP induced by using K^2^ stimulation electrodes has a potential to provide a link between subjective pain estimation and objective electrophysiological measures. Furthermore, it has been shown that both the amplitude of the PCES-EP as well as the induced pain intensity has a high test-retest reliability [31].

The aim of the study was to investigate the capability of PCES-EP to objectively evaluate the CPM-effect electrophysiologically. Therefore, we applied PCES as TS and recorded both the subjective pain ratings induced by PCES-EP, and the amplitude of PCES-EP at baseline, during and after CS. As CS we chose the immersion of the hand into cold water as it is commonly used and elicits stronger CPM-effects compared to other CS [32] and has been also shown to be a reliable stimulus in terms of the induced pain intensity [33].

## Methods

### Participants

After approval by the local ethics committee (Reg. Nr. 15–5300; 06–16-2015) 18 healthy subjects (age > 18 years) were recruited after informed consent, using recently recommended inclusion and exclusion criteria [34]. The study was conducted in the department of Neurology, University Hospital Bergmannsheil Bochum, Germany between June and August 2015. Exclusion criteria included current pain, neuropathy, nerve lesions, topical drug treatment, history of neurological, psychiatric or severe cardiovascular diseases. A sample size calculation was performed using data on changes of the N2-P2-amplitudes of contact heat evoked potentials (CHEP) from a study examining the conditioned pain modulation with CHEP as a test stimulus and painful heat as conditioning stimulus [2] and revealed for a power of 80%, type I error of 0.05 and an estimated drop-out rate of 5% a sample size of at least 14 subjects per study arm in a crossover design.

Before stimulation we determined handedness by the German version of the Edinburgh Handedness Inventory [35].

### PCES-EP

We denoted the pain perceived during painful cutaneous electrical stimulation of the intraepidermal nociceptive fibers for PCES-EP-recording as ‘*PCES pain*’. ‘*PCES-EP -amplitude*’ was the corresponding peak-to-peak amplitude N1-P1 recorded over central electrode Cz according to the international 10–20-System.

**Fig. 1:**
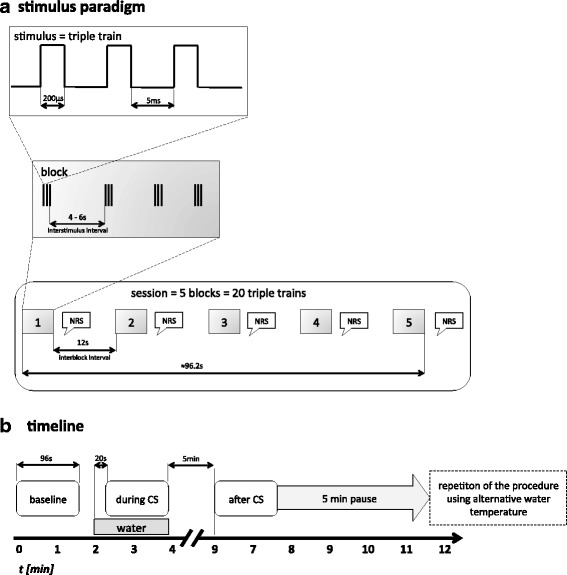
Stimulation paradigm (**a**) and timeline of experimental procedure (**b**)

For PCES, we placed three custom-built concentric surface electrodes (K^2^ electrodes used to induces pain-related evoked potentials (PREP) in previous studies) [36] on the dominant hand in a triangular formation (distance of 1.5–2 cm at the dorsum of the hand, supply area of the superficial radial nerve). The stimulation electrodes are connected in parallel to a stimulator (Digitimer DS7A). We fixed the electrodes as such that all of the stimuli were applied at the same location. One electrical stimulus applied simultaneously by all three stimulation electrodes consisted of a train of three monopolar squares waves of 200 μs duration with 5 ms interval in between (see Fig. 1). We applied 20 triple trains in 5 blocks with a variable intertrain interval of 4–6 s in a pseudorandomized manner, with a fixed interblock interval of 12 s. Thus, each of the 5 blocks consisted of 4 triple trains à 10.6 ms and lasted 15.04 s, thus the whole procedure with application of 20 triple trains lasted about 96.17 s.

After every 4 triple trains subjects reported the current PCES-pain intensity to the examiner as a rating on the 101-point numerical rating scale (NRS, 0 = no pain and 100 = strongest pain imaginable) after they have been instructed and familiarized with the NRS prior to the experiment, thus resulting in a total of 5 ratings per PCES-EP-session. As the 5 values within the baseline period were stable (data not shown), the PCES pain intensity was defined as the mean of the 5 ratings for further statistics.

According to the international 10–20-System, the recording electrode was at Cz, referred to linked earlobes (A1 and A2). Impedances were kept below 5 kΩ. PCES-EPs were recorded and stored for offline analysis with a 32-channel-amplifier (Brain Amp, Brain Products, Germany; bandwidth: 1 Hz-1 kHz, sampling rate: 5 kHz). PCES-EPs were analyzed in sweeps from 200 ms before and 800 ms after onset of the triple pulses, and averaged. We determined the latency of N1-peak and N1-P1 peak-to-peak amplitude. In accordance to previous studies [11, 29, 30], we rejected the first sweep to avoid contamination by startle response. Prior to averaging, one experienced investigator evaluated all records individually to exclude technical or blink artifacts.

### Experimental procedure

The study was designed as a randomized placebo-controlled crossover study.

During the experiment subjects sat in a comfortable chair in an air-conditioned room.

The conditioned pain modulation was assessed using the PCES as TS. As CS we used immersion of the non-dominant hand into a container filled with cold water at a temperature of 10 ± 2 °C (cold and intended to be painful), or – as a control condition – water of 24 ± 2 °C (cold, but intended to be non-painful), in a crossover design. Participants were randomized via a paper-pencil-based lot to sequence A (first 24 °C cold water; then 10 °C) or to sequence B (vice versa). During the whole experiment, an infrared thermometer measured the skin temperature next to the used K^2^-stimulation electrodes before and after every PCES-EP-session.

The CPM-assessment consisted of three blocks denoted as ‘baseline, ‘during CS and ‘after CS. At first, subjects were familiarized with the stimulus characteristic by applying current intensities inducing subthreshold (i.e. painless tingling) and suprathreshold (i.e. pinprick-like pain) sensations. Then, the ‘baseline’-block started with a PCES-EP -session to receive stable PCES-EP and stable PCES-pain corresponding to NRS 40–60, estimating the pain intensity after every 4 electrical stimuli. Therefore, stimulus intensity of presented triple pulses was adjusted until subjects perceived a stable electrically-induced pain level of 5 successive stimuli. We defined the pain intensity as stable, if the value of four successive pain ratings did not change more than 5 points on the NRS (0–100). In the ‘during CS’-block subjects immersed their non-dominant hand into 24 °C or 10 °C cold water, depending on the randomization. An investigator (OH) not involved in the documentation of pain and analysis of PCES-EP prepared the conditioning stimulus (temperature of cold-water) and generated the random allocation sequence. The second examiner (ÖÖ) was blinded to the intervention. Additionally, the subjects were blinded for the aim of the experiments.

Immediately after hand immersion into the water, subjects estimated the pain intensity induced by the coldness on the NRS (0–100). After 20 s, the PCES-EP recordings were repeated during CS, and after every 4 stimuli the subjects estimated the pain intensity both induced by electrical stimulation and cold water. Finally, after about 2 min, subjects took their hand out of the water. Five min later, the PCES-EP-session ‘after CS’ was performed. After additional 5 min, the procedure was repeated using the alternative water temperature, thus the interval between the end of the conditioning stimulus of the first test session and the begin of the testing stimulus of the second test session was 10 min, as previously recommended [12].

#### Definition of the CPM-effect

The CPM-effect_PAIN_ was calculated as difference between the mean of the 5 pain ratings during CS and the mean of the 5 pain ratings at baseline (each after electrical stimulus No. 4, 8, 12, 16 and 20) [5, 37].

***CPM-effect***_***PAIN***_ *= Mean of 5 pain ratings (during) – Mean of 5 pain ratings (baseline).*

Additionally, we calculated the CPM-effect_AMPLITUDE_ as difference between the mean of the 19 PCES-EP-amplitudes during CS and the mean of the 19 PCES-EP-amplitudes at baseline:

***CPM-effect***_***AMPLITUDE***_ *= Mean of 19 PCES-EP-amplitudes (during) – Mean of 19 PCES-EP-amplitudes (baseline).*

According to Granot, Yarnitsky et al., every difference < 0 was suggested to represent an efficient pain inhibition [5, 37], both for CPM-effect_PAIN_ and CPM-effect_AMPLITUDE_. However, others have suggested that some allowance should be made for measurement error, though using another CPM-protocol, based on pressure pain thresholds as test stimulus [38]. Yet, the current recommendations for CPM-practice stated no cut-off value for definition of a sufficient CPM-effect [12].

### Statistical analysis

All analyses were conducted using SPSS version 19 (IBM, New York, NY), with statistical significance at *p* < 0.05.

Descriptive statistics are presented as means and standard deviations. Nominal parameters were analyzed using χ2-tests.

ANOVA for repeated measures was performed for the dependent variables PCES-EP-amplitude, PCES-pain, and PCES-EP-N1-latency with between-subjects factors group of subjects (sequence A and B), levels of “temperature levels” (24 °C and 10 °C water temperature), and within-subjects factor “time period” (baseline, during and after cold water stimulation).

Differences in the CPM-effect based on PCES-pain and PCES-EP-amplitude between both temperature conditions (10 °C-water and 24 °C-water as CS) were evaluated using two-tailed paired t-tests. Pearson correlation analysis was performed between PCES-EP-amplitude and PCES-pain, between their changes during CPM-assessment (based on the difference, i.e. CPM-effect_PAIN_ as well as CPM-effect_AMPLITUDE_, and based on the ratios for pain intensity and amplitude of the evoked potentials, i.e. “during CS” divided by “baseline”). Further on, the changes during CPM-assessment were correlated to the cold water pain intensity. Additionally, linear regression analysis was performed with ‘changes of PCES-EP-amplitude’ as dependent variable, and ‘changes of PCES-pain’ and ‘pain induced by the cold water’ as independent variables.

## Results

### Participants

One subject failed to perceive pain with a reproducible intensity of 40–60 on the NRS during TS application, and was therefore excluded. Hence, 17 participants (age: 28.1 ± 2.1 years (mean ± SEM), range 19–45 years; 9 female) were included from further analysis. Eight subjects were allocated to sequence A (firstly exposed to 24 °C cold water; then to 10 °C) and 9 to sequence B (firstly exposed to 10 °C cold water; then to 24 °C). There were no unintended side effects during the experiments.

### Stimulation intensity

The mean stimulus intensity to induce a pain of NRS 50–60 was 7.3 ± 5.4 mA (range 1.6 – 20 mA). Please note that in our stimulation setting all 3 electrodes were connected in parallel to a single stimulator. Hence, the given current intensities are proportionally distributed over all electrodes. The stimulation was well tolerated without evoking any muscle twitches, and no one quit study participation due to intolerably painful stimuli.

### CPM-effect

**Fig. 2:**
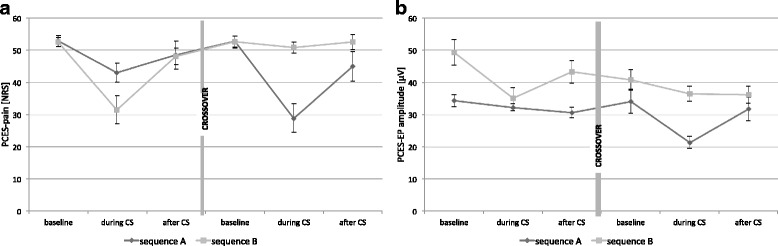
Time course of PCES-pain (**a**) and PCES-EP-amplitude (**b**) in sequence A (firstly 24 °C cold water; then 10 °C cold water as conditioning stimulus) and B (firstly 10 °C cold water; then 24 °C cold water as conditioning stimulus)

N1-P1-amplitude, N1- and P1-latency of PCES-EP were normally distributed according to the Kolmogorov–Smirnov test and visual inspection of data histograms. Both for PCES-pain and PCES-EP-amplitude, ANOVA yielded a significant effect of the factor “time period” (PCES-pain: F_2;60_ = 35.212, *p* < 0.001; PCES-EP-amplitude: F_2;60_ = 42.423, *p* < 0.001) and a significant effect of “temperature level” (PCES-pain: F_2;60_ = 12.686, *p* < 0.001; PCES-EP-amplitude: F_2;60_ = 21.404, *p* < 0.001), while there was no interaction between “time period” and “temperature level” (PCES-pain: F_2;60_ = 0.319, *p* = 0.728; PCES-EP-amplitude: F_2;60_ = 0.529, *p* = 0.592). We found a significant effect of “sequence” on the PCES-EP-amplitude (F = 11.843, *p* < 0.005), probably due to differences in the amplitudes between both subject populations, at the aimed pain intensity of NRS 60 on the 101-point rating scale (see Fig. 2b). However, there was no significant effect of “sequence” on the pain intensity (F_2;60_ = 1.561, *p* = 0.221), and more importantly, there was no significant interaction of “time period” and “sequence” (PCES-pain: F_2;60_ = 1.297, *p* = 0.281; PCES-EP-amplitude: F_2;60_ = 0.870, *p* = 0.424, Fig. 2). Thus, the CPM-conditions were analyzed independently from the sequence.

For N1-latency, ANOVA yielded a significant effect of the factor “time period” (F_2;60_ = 3.568, *p* = 0.034), but there was no effect of “sequence”, “temperature level” and interaction of “time period” and “sequence”. Independent of factor “sequence” and “temperature” level N1-latency increased significantly from “baseline” (131.5 ± 11.2 ms) to “during CS” (133.7 ± 12.2 ms; *P* = 0.025), while there was no change from “during CS” to “after CS” (133.9 ± 12.1 ms; *P* = 0.823).

### Skin temperature

ANOVA for repeated measurements yielded neither significant skin temperature changes during the experiment (factor “time period”: F_2;64_ = 0.142, *p* = 0.868), nor significant differences between the 10 °C- and 24 °C-conditions (factor “temperature level”: F_1;32_ = 0.037, *p* = 0.849), and no significant interaction between both factors (F_2;64_ = 0.457, *p* = 0.635).

### *Cold water pain, PCES-pain, and* PCES-EP*-amplitude during CPM-assessment*

In the 10 °C-condition the average rating of the cold water pain was significantly higher (58.9 ± 25.4, range: 0–90.8 NRS), compared to the 24 °C-condition (6.9 ± 1.8, range: 0–41.7 NRS, *p* < 0.001).

**Fig. 3:**
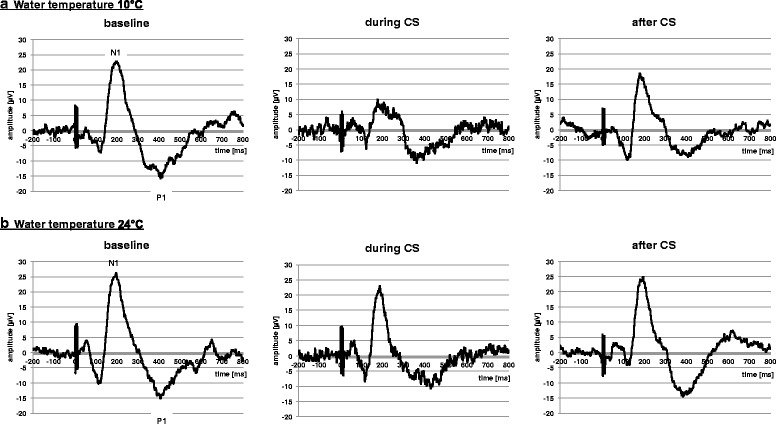
PCES-EPs of one participant shown at baseline, during CS, and after CS in both conditions: **a**) water temperature 10 °C, and **b**) water temperature 24 °C

In the 10 °C-condition, both PCES-EP-amplitude (*p* = 0.003) and PCES-pain (*p* < 0.001) decreased between the ‘baseline’ and ‘during CS’ session (Table 1). After CS-termination, both PCES-pain (*p* < 0.001) and PCES-EP-amplitude (*p* = 0.022) increased. In the 24 °C-condition only PCES-pain decreased significantly ‘during CS’ compared to ‘baseline’ (*p* = 0.019), but not PCES-EP-amplitude (*p* = 0.194) (see Fig. 3). After CS-termination we found no changes of both parameters (PCES-EP-amplitude *p* = 0.678; PCES-pain *p* = 0.256).

**Table 1:** Parameters of the Conditioned Pain Modulation (CPM)

	10° condition			24° condition		
	PCES-induced pain (NRS 0–100)	PCES- amplitude (μV)	PCES-latency (ms)	PCES-induced pain (NRS 0–100)	PCES-amplitude (μV)	PCES-latency (ms)
baseline	52.6 ± 4.4	42.1 ± 13.4	130.7 ± 10.5	52.7 ± 5.0	37.8 ± 8.3	132.2 ± 12.1
during CS	30.3 ± 12.5	28.7 ± 10.5	133.5 ± 11.9	47.1 ± 7.8	34.5 ± 5.8	133.9 ± 12.8
after CS	46.6 ± 10.4	37.9 ± 11.7	133.3 ± 12.8	50.6 ± 9.6	33.6 ± 7.1	134.5 ± 11.8
CPM-effect (abs.)	−22.4 ± 13.6	−13.5 ± 6.4	2.8 ± 6.2	−5.6 ± 6.5	−3.3 ± 4.7	1.6 ± 4.8
CPM-effect (rel.)	42.0 ± 24.3%	32.0 ± 10.7%	−2.1 ± 5.1%	10.5 ± 12.6%	7.2 ± 11.0%	−1.3 ± 3.7

Regarding the PCES-pain ratings, 16 subjects (94%) had a CPM-effect_PAIN_ < 0 in the 10 °C-condition versus 12 subjects (71%) in the 24 °C-condition (*p* = 0.072). Regarding the PCES-EP-amplitudes, all subjects had a CPM-effect_PCES_ < 0 in the 10 °C-condition versus 13 subjects (76%) in the 24 °C-condition (*p* = 0.003). However, both CPM-effect_PAIN_ and CPM-effect_AMPLITUDE_ were significantly higher in the 10 °C-condition than in the control condition (both *p* < 0.00001, Table 1).

CPM-assessment based on the PCES-EP-amplitude (N1-P1) and PCES-EP-latency (N1), as well as the PCES-pain; CPM-effects are shown for absolute (abs.) and relative (rel.) changes from baseline to during CS; data was accumulated disregarding the randomization sequence (sequence A or B), as there was no evidence for a sequence-effect on the results.

### Relation between cold water pain, PCES-pain and PCES-EP-amplitude during CPM-assessment

**Fig. 4:**
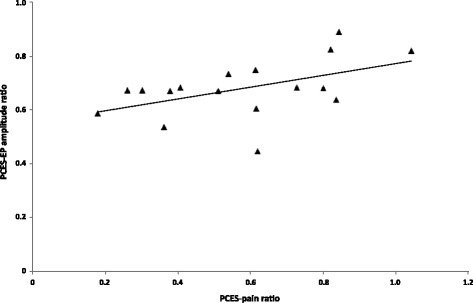
Changes of PCES-EP amplitude and changes of PCES-pain in subjects in the 10° cold water condition, expressed as ratios (during CS/ baseline)

In the 10 °C-condition, we assessed the interaction between cold water pain, changes of PCES-EP-amplitudes and changes of PCES-pain. Analyzing the changes of PCES-pain and PCES-EP-amplitude as ratios (‘during CS’/‘baseline’), the ratios for PCES-pain and PCES-EP-amplitude correlated significantly (*r* = 0.50, *p* < 0.05, Fig. 4).

The cold water pain intensity correlated with the PCES-EP-amplitude ratios (*r* = −0.5, *p* < 0.05) and with the PCES-pain ratios (*r* = −0.6, *p* < 0.01). PCES-EP-latency correlated with PCES-pain (*r* = −0.65; *p* < 0.01), but not with cold pain (*r* = 0.05; *p* = 0.429) or PCES-EP-amplitude (*r* = −0.2; *p* = 429).

## Discussion

Applying PCES using K^2^ stimulation electrodes as TS, we could induce endogenous pain inhibition by hand immersion in 10 °C cold water, and assessed the CPM-effect both subjectively based on pain ratings, and objectively using electrophysiological measures. On a group level, during the 10 °C-condition, the PCES-induced pain and the PCES amplitude was reduced on average by 42% and 32%, respectively (vs. 10% and 7%, respectively, during the control condition). On single-subject level, the PCES-EP-amplitude decreased in all subjects and the PCES-pain intensity decreased in 94% of the cases, in contrast to other published protocols, which were not able to induce a CPM-effect in all subjects, e.g. [23, 38]. More important, the reported changes in subjective pain intensity and the objectively measured changes in PCES-EP-amplitude correlated significantly. On a group level, the changes in both subjective pain ratings and objective electrophysiological measures were significant only in the painful water condition, but not in the control condition where the PCES-EP-amplitude did not change significantly.

### PCES as TS

The magnitudes of this CPM-effect based on subjective pain ratings during PCES in our study (42 ± 24%) is comparable to previous studies using painful cold as CS, where the CPM-effect based on subjective pain ratings varied between 10% and 60% from the baseline [13, 39, 40].

We investigated the feasibility of PCES-pain to assess the CPM-effect as this technique provides several advantages over other TS. The very confined stimulation zone of K^2^ stimulation electrodes (3 × 19.6 mm^2^ = 58.8 mm^2^) enables stimulation of small skin areas, whereas heat as most often used TS is usually being applied via thermodes with a contact area of 900mm^2^. Another advantage of PCES using K^2^ stimulation electrodes is the brief, distinct and well-tolerable pinprick-like pain. The electrical stimuli are safe and can be repeated without relevant risk of tissue damage. While using repeated heat pain as TS is limited due to habituation of both pain ratings and contact heat evoked potentials (CHEP) [41, 42], in our study the PCES-pain intensity showed no habituation and remained stable over 20 stimuli with pseudorandomized intertrain intervals of 4–12 s (data not shown). Thus, CPM-paradigms using heat pain as TS need longer interstimulus intervals to prevent habituation and longer latencies to raise thermode temperature from neutral skin temperature (~32 °C) to painful heat (~43°-47 °C). In contrast, using concentric electrodes to elicit PCES-EP enable a reliable judgment of pain intensity and a quick adjustment to target pain intensity by modifying current intensity [31].

### CPM-effect on the PCES-EP-amplitude and PCES-EP-N1-latency

In our CPM-paradigm, the PCES-EP-amplitude significantly decreased during CS by 32 ± 11%, which was much more pronounced than CHEP-amplitudes [2] or SEP-amplitudes after chemonasal CO_2_-stimulation [22] as TS. Furthermore, the PCES-EP-amplitude is comparable to studies assessing SEP-amplitudes after CO_2_ laser stimulation [17], and after electrical tooth stimulation [20, 21].

There was a significant, yet moderate correlation between the changes in PCES-EP-amplitudes and PCES-pain intensity. A correlation between the decrease of EP amplitudes and the pain intensity has been also reported, albeit lower, for CPM-models using CHEP as TS and hot water pain as CS [2], and SEP after CO_2_-laser stimulation as TS and heat as CS (no detailed data presented) [18]. CHEP reflect C-fiber transmission, whereas pinprick-like pain during cortically recorded so-called pain-related evoked potentials (PREP) is assumed to represent mainly A-delta-fiber function [25, 29], though the latter type of stimulation cannot exclude the possibility of also low-threshold mechanoreceptors being involved. Thus, the interpretation of differences in the effects of both techniques needs to be done with care. A study recording SEP after electrical tooth stimulation as TS and CO_2_-laser stimulation as CS have also found a significant CPM-effect for both pain intensity and EP-amplitudes, without presenting any data on their correlation [17].

Another study investigated CPM using chemo-somatosensory evoked potentials (CSSEPs) elicited by CO_2_-gas as TS and tonic heat pain as CS, and found significantly reduced CSSEP-amplitudes and prolonged latencies during CS [22]. The authors postulated that earlier CSSEP-components reflect the sensory input, while later components reflect the cerebral signal processing, and based on their findings they concluded the analgesic effects of CPM at a cerebral level. Although the local origin of PCES-EP-components was not investigated, previous PREP-studies indicated that the investigated N1-component reflects an early cortical processing [29, 30]. Therefore, the CPM-induced reduction of PCES-EP-amplitudes in our study mainly refers to modulation of the sensory input. In contrast to our results, CPM-induced changes of CSSEP were not reflected by the subjects’ pain ratings [22].

Based on previous studies, PCES-EP induced by K^2^ stimulation electrodes are recorded after activation of intraepidermal nociceptive fibers [27, 29, 30]. The concentric design and small anode–cathode distance of K^2^ stimulation electrodes produce high current density at low current intensities, resulting predominantly in stimulation of nociceptive Aδ-fibers [11, 36]. Furthermore, the well-localized, sharp, pin-prick like sensation and the conduction velocity measured corresponds to Aδ-fiber activation [43].

Results of functional magnetic resonance imaging studies demonstrate that PCES induces activation within the pACC (posterior part of the anterior cingulate cortex), PCC (posterior cingulate cortex), insula, and somatosensory cortex (SII) [11].

Here, an increase of PCES stimulus intensity resulted in increasing pain ratings, increasing PCES-EPs and increasing BOLD response within these brain regions [11]. Although PCES-EP showed a high test-retest-reliability concerning the commonly used parameters N1-latency and N1P1-amplitude [31], the present study demonstrates that PCES-induced pain and PCES-EPs can be modulated by CPM.

To this point, it remains open which targets in the sensory pathway are mainly involved. Using a different kind of electrical skin stimulus, Bromm et al. demonstrated that the opioid agonist tilidin is able to reduce pain ratings and evoked potentials [44].

Based on the concept of the diffuse noxious inhibitory controls (DNIC), an activation of the spino-bulbo-spinal mechanism or descending pathways are of major importance to modulate pain perception by inhibition or facilitation of noxious information [2, 45]. Further studies are required to identify the exact link between the amplitudes of PCES-EPs and pain perception. Here, a combination of CPM paradigms including PCES-EPs recorded at a cortical level and nociceptive flexion reflex responses evaluating mainly the spinal modulation may be beneficial to explore more detailed the exact mechanisms of the endogenous analgesia.

Furthermore, we analyzed the changes of N1-latency of cortical PCES-EP during CPM. We found a significant increase of N1-latency only from ‘baseline’ to ‘during CS’ application. This is in accordance to previous studies using chemo-somatosensory evoked potentials as test stimulus and tonic heat pain as conditioning stimulus to induce CPM [22]. Interestingly, in both conditions of 10° and 24°cold water as CS, the N1-latency increased. However, some subjects perceived the 24 °C cold water which was intended to be a non-painful control CS, also as a painful, similarly to another study using a conditioning stimulus of 25 °C, which was also rated as painful on an NRS on average by 2.3 ± 0.4 [46]. Hence, this may explain the missing difference between both conditions.

It should be noted that a CPM-effect was in fact elicited in 71–76% (depending on the outcome measure) of participants in the control condition, though it was to a lesser extent than in the 10 °C condition. Similarly to Treister et ale46], examining a CPM model with conditioning stimulus of 25 °C, where a mean CPM-effect amounted to 22% (in our study 10.5 ± 12.6%). Such non-noxious inhibitory control-like effects have already been previously discussed as potentially representing the amount of habituation or psychological effects, like anxiety-related increased pain at the beginning of a series of experimental trials [46].

### Relation between cold water pain and CPM-magnitude

The cold water pain intensity induced significantly changes of both PCES-pain and PCES-EP-amplitudes. The more painful the subjects rated the cold water (as conditioning stimulus), the less painful they rated the PCES-pain during CS and the smaller were the PCES-EP-amplitudes during CS. This is in contrast to previous results, where the magnitude of the CPM-effect, using hot water hand immersion as CS and contact heat pain as TS, depended only from the CS-temperature, but not from the perceived CS-pain intensity [47]. However, our results are consistent with another study showing that pain inhibition under CPM seems to depend on the perceived level of the CS pain rather than solely its physical intensity [48]. Interestingly, the cold water pain intensity showed no effect on PCES-EP latency, suggesting that the CPM-effect based on the electrophysiological response reflects different central processing compared to the CPM-effect based on subjective pain ratings. Further studies are needed to explore this issue.

### Limitations

Some limitations must be considered when interpreting the findings of the present study. Despite randomization of participants there was a difference of the initial N1-P1 amplitudes of PCES-EP between group A and B. This might result from the interindividual variation of PCES-EP-amplitudes, as our study was powered to detect differences between the PCES-EP-amplitudes before and after application of a conditioning stimulus. Considering this, we analyzed changes of amplitudes and pain ratings during the CPM by calculating ratios in addition to the calculated differences (representing the CPM-effect as per definition recommended) to avoid an impact of the individual variation of the amplitude on the results.

Another possible limitation is the fact that, retrospectively, subjects perceived the difference in temperature between both immersions of the hand. However, they were blinded for the aim of the study. Also, we found no carry over effect, suggesting that the limitation on blinding the water temperature to the subjects is not relevant for the study. As discussed above, during the non-painful control condition about 50% of the subjects reported painful perception after immersion in painful 24 °C cold water, here, about 20% estimated pain levels above 10 of NRS. Using a higher temperature as a neutral control condition could have reduced pain perception and, hence, a comparison of distinct painful and non-painful conditions may have result in even greater differences between the CPM-effect induced by both different CS. Taken this into account, we assessed the interaction between cold water pain, changes of PCES-EP-amplitudes and changes of PCES-pain independently from the CPM-condition (see above). Though, it should be mentioned that calculating a Pearson correlation based on 24 datasets where the CS was rated as painful (see results section), represents a statistical limitation.

In a previous study the CPM-effect based on pain intensity after electrical stimulation, induced by cold pressor test showed acceptable reliability [1]. However, results regarding the re-test reliability of different CPM testing protocols vary widely between studies, and seem to depend on the used CS and TS as well as the time interval (for discussion see Gehling et al. 2016 [33]), therefore the reliability of the introduced CPM model here should be further investigated. Nevertheless, the used TS and CS were shown to be reliable, in terms of the induced pain intensity (for TS and CS) and evoked potentials [31, 33].

Also, some limitations regarding the selective activation of nociceptive fibers during electrical stimulation of the used concentric electrodes must be admitted. Recent studies have questioned whether such electrical stimulation through specially designed electrodes selectively activates nociceptive A-delta fibers [49]. Furthermore, different stimulation intensities and electrode designs (resulting in different spreading of current due to different resistances during intracutan or surface stimulation) make a direct comparison impossible. Further studies are needed to clarify this issue and to examine the underlying mechanisms of reduction of the PCES-EP-amplitude and N1-latency during painful CS application.

There is inconsistent data about the influence of menstrual cycle on CPM [50–53]. In the present study, we did not control for the stage of menstrual cycle of female participants. This might be seen as a limitation of the study, however, detecting such differences was not in the scope of the present study and as both CPM assessments were performed on the same day, differences in the results between both conditions due to menstrual cycle can be excluded.

## Conclusions

In summary, we present a novel CPM-paradigm being able to induce a significant decrease of the subjective pain intensity in healthy subjects by hand immersion in painful cold water. More importantly, and to our knowledge for the first time, we were able to demonstrate a concomitant decrease of the PCES-EP-amplitude during CPM and the perceived intensity of the PCES induced pinprick-like pain, which both correlated with the pain intensity of the conditioning stimulus. Thus, this CPM model is a feasible and cost-effective novel method to objectify and evaluate interventions related to the function of endogenous pain modulation.

## Abbreviations

CHEP: Contact heat evoked potential
CPM: Conditioned pain modulation
CS: Conditioning stimulus
CSSEP: Chemo-somatosensory evoked potential
DNIC: Diffuse noxious inhibitory controls
NRS: Numerical rating scale
pACC: Posterior part of the anterior cingulate cortex
PCC: Posterior cingulate cortex
PCES: Painful cutaneous electrical stimulation
PCES-EP: PCES-evoked potential
PREP: Pain-related evoked potential
SEP: Sensory evoked potential
SII: Secondary somatosensory cortex
TS: Test stimulus

## References

[1] Biurrun Manresa JA, Fritsche R, Vuilleumier PH, Oehler C, Morch CD, Arendt-Nielsen L, Andersen OK, Curatolo M (2014). Is the conditioned pain modulation paradigm reliable? A test-retest assessment using the nociceptive withdrawal reflex. PLoS One.

[2] Moont R, Crispel Y, Lev R, Pud D, Yarnitsky D (2011). Temporal changes in cortical activation during conditioned pain modulation (CPM), a LORETA study. Pain.

[3] Nahman-Averbruch H, Yarnitzky D, Granovsky Y, Gerber E, Dagul P, Granot M (2013). The role of stimulation parameters on the conditioned pain modulation response. Scand J Pain.

[4] Lewis GN, Rice DA, McNair PJ (2012). Conditioned pain modulation in populations with chronic pain: a systematic review and meta-analysis. J Pain.

[5] Yarnitsky D, Granot M, Nahman-Averbuch H, Khamaisi M, Granovsky Y (2012). Conditioned pain modulation predicts duloxetine efficacy in painful diabetic neuropathy. Pain.

[6] Grosen K, Vase L, Pilegaard HK, Pfeiffer-Jensen M, Drewes AM (2014). Conditioned pain modulation and situational pain catastrophizing as preoperative predictors of pain following chest wall surgery: a prospective observational cohort study. PLoS One.

[7] Ram KC, Eisenberg E, Haddad M, Pud D (2008). Oral opioid use alters DNIC but not cold pain perception in patients with chronic pain - new perspective of opioid-induced hyperalgesia. Pain.

[8] Petersen KK, Graven-Nielsen T, Simonsen O, Laursen MB, Arendt-Nielsen L (2016). Preoperative pain mechanisms assessed by cuff algometry are associated with chronic postoperative pain relief after total knee replacement. Pain.

[9] Youssef AM, Macefield VG, Henderson LA (2016). Pain inhibits pain; human brainstem mechanisms. NeuroImage.

[10] Bogdanov VB, Vigano A, Noirhomme Q, Bogdanova OV, Guy N, Laureys S, Renshaw PF, Dallel R, Phillips C, Schoenen J (2015). Cerebral responses and role of the prefrontal cortex in conditioned pain modulation: an fMRI study in healthy subjects. Behav Brain Res.

[11] Obermann M, Pleger B, de Greiff A, Stude P, Kaube H, Diener HC, Katsarava Z (2009). Temporal summation of trigeminal pain in human anterior cingulate cortex. NeuroImage.

[12] Yarnitsky D, Bouhassira D, Drewes AM, Fillingim RB, Granot M, Hansson P, Landau R, Marchand S, Matre D, Nilsen KB (2015). Recommendations on practice of conditioned pain modulation (CPM) testing. Eur J Pain.

[13] Pud D, Granovsky Y, Yarnitsky D (2009). The methodology of experimentally induced diffuse noxious inhibitory control (DNIC)-like effect in humans. Pain.

[14] Piche M, Arsenault M, Rainville P (2009). Cerebral and cerebrospinal processes underlying counterirritation analgesia. J Neurosci.

[15] Sandrini G, Ruiz L, Capararo M, Danilov A, Beretta A, Nappi G (1993). Effects of dothiepin on nociceptive flexion reflex and diffuse noxious inhibitory controls in humans. Eur J Pharmacol.

[16] Drummond PD (2003). The effect of trigeminal nociceptive stimulation on blink reflexes and pain evoked by stimulation of the supraorbital nerve. Cephalalgia.

[17] Fujii-Abe K, Oono Y, Motohashi K, Fukayama H, Umino M (2010). Heterotopic CO2 laser stimulation inhibits tooth-related somatosensory evoked potentials. Pain Med.

[18] Kakigi R (1994). Diffuse noxious inhibitory control. Reappraisal by pain-related somatosensory evoked potentials following CO2 laser stimulation. J Neurol Sci.

[19] Watanabe S, Kakigi R, Hoshiyama M, Kitamura Y, Koyama S, Shimojo M (1996). Effects of noxious cooling of the skin on pain perception in man. J Neurol Sci.

[20] Motohashi K, Umino M (2001). Heterotopic painful stimulation decreases the late component of somatosensory evoked potentials induced by electrical tooth stimulation. Brain Res Cogn Brain Res.

[21] Motohashi K, Umino M, Fujii Y (2002). An experimental system for a heterotopic pain stimulation study in humans. Brain Res Brain Res Protoc.

[22] Kunz M, Mohammadian P, Renner B, Roscher S, Kobal G, Lautenbacher S (2014). Chemo-somatosensory evoked potentials: a sensitive tool to assess conditioned pain modulation?. Somatosens Mot Res.

[23] Kennedy DL, Kemp HI, Ridout D, Yarnitsky D, Rice AS (2016). Reliability of conditioned pain modulation: a systematic review. Pain.

[24] Mueller D, Obermann M, Koeppen S, Kavuk I, Yoon MS, Sack F, Diener HC, Kaube H, Katsarava Z (2010). Electrically evoked nociceptive potentials for early detection of diabetic small-fiber neuropathy. Eur J Neurol.

[25] Obermann M, Katsarava Z, Esser S, Sommer C, He L, Selter L, Yoon MS, Kaube H, Diener HC, Maschke M (2008). Correlation of epidermal nerve fiber density with pain-related evoked potentials in HIV neuropathy. Pain.

[26] Uceyler N, Kahn AK, Kramer D, Zeller D, Casanova-Molla J, Wanner C, Weidemann F, Katsarava Z, Sommer C (2013). Impaired small fiber conduction in patients with Fabry disease: a neurophysiological case-control study. BMC Neurol.

[27] Uceyler N, Zeller D, Kahn AK, Kewenig S, Kittel-Schneider S, Schmid A, Casanova-Molla J, Reiners K, Sommer C (2013). Small fibre pathology in patients with fibromyalgia syndrome. Brain.

[28] Bromm B, Meier W (1984). The intracutaneous stimulus: a new pain model for algesimetric studies. Methods Find Exp Clin Pharmacol.

[29] Katsarava Z, Ayzenberg I, Sack F, Limmroth V, Diener HC, Kaube H (2006). A novel method of eliciting pain-related potentials by transcutaneous electrical stimulation. Headache.

[30] Oh KJ, Kim SH, Lee YH, Kim JH, Jung HS, Park TJ, Park J, Shinn JM (2015). Pain-related evoked potential in healthy adults. Ann Rehabil Med.

[31] Ozgul OS, Maier C, Enax-Krumova EK, Vollert J, Fischer M, Tegenthoff M, Hoffken O (2017). High test-retest-reliability of pain-related evoked potentials (PREP) in healthy subjects. Neurosci Lett.

[32] Oono Y, Nie H, Matos LM, Wang K, Arendt-Nielsen L (2011). The inter- and intra-individual variance in descending pain modulation evoked by different conditioning stimuli in healthy men. Scand J Pain.

[33] Gehling J, Mainka T, Vollert J, Pogatzki-Zahn EM, Maier C, Enax-Krumova EK (2016). Short-term test-retest-reliability of conditioned pain modulation using the cold-heat-pain method in healthy subjects and its correlation to parameters of standardized quantitative sensory testing. BMC Neurol.

[34] Gierthmuhlen J, Enax-Krumova EK, Attal N, Bouhassira D, Cruccu G, Finnerup NB, Haanpaa M, Hansson P, Jensen TS, Freynhagen R (2015). Who is healthy? Aspects to consider when including healthy volunteers in QST-based studies- a consensus statement by the EUROPAIN and NEUROPAIN consortia. Pain.

[35] Oldfield RC (1971). The assessment and analysis of handedness: the Edinburgh inventory. Neuropsychologia.

[36] Kaube H, Katsarava Z, Kaufer T, Diener H, Ellrich J (2000). A new method to increase nociception specificity of the human blink reflex. Clin Neurophysiol.

[37] Granot M, Weissman-Fogel I, Crispel Y, Pud D, Granovsky Y, Sprecher E, Yarnitsky D (2008). Determinants of endogenous analgesia magnitude in a diffuse noxious inhibitory control (DNIC) paradigm: do conditioning stimulus painfulness, gender and personality variables matter?. Pain.

[38] Locke D, Gibson W, Moss P, Munyard K, Mamotte C, Wright A (2014). Analysis of meaningful conditioned pain modulation effect in a pain-free adult population. J Pain.

[39] Edwards RR, Ness TJ, Weigent DA, Fillingim RB (2003). Individual differences in diffuse noxious inhibitory controls (DNIC): association with clinical variables. Pain.

[40] Baad-Hansen L, Poulsen HF, Jensen HM, Svensson P (2005). Lack of sex differences in modulation of experimental intraoral pain by diffuse noxious inhibitory controls (DNIC). Pain.

[41] Agostinho CM, Scherens A, Richter H, Schaub C, Rolke R, Treede RD, Maier C (2009). Habituation and short-term repeatability of thermal testing in healthy human subjects and patients with chronic non-neuropathic pain. Eur J Pain.

[42] Greffrath W, Baumgartner U, Treede RD (2007). Peripheral and central components of habituation of heat pain perception and evoked potentials in humans. Pain.

[43] Katsarava Z, Yaldizli O, Voulkoudis C, Diener HC, Kaube H, Maschke M (2006). Pain related potentials by electrical stimulation of skin for detection of small-fiber neuropathy in HIV. J Neurol.

[44] Bromm B, Seide K (1982). The influence of tilidine and prazepam on withdrawal reflex, skin resistance reaction and pain rating in man. Pain.

[45] Sprenger C, Bingel U, Buchel C (2011). Treating pain with pain: supraspinal mechanisms of endogenous analgesia elicited by heterotopic noxious conditioning stimulation. Pain.

[46] Treister R, Eisenberg E, Gershon E, Haddad M, Pud D (2010). Factors affecting - and relationships between-different modes of endogenous pain modulation in healthy volunteers. Eur J Pain.

[47] Nir RR, Granovsky Y, Yarnitsky D, Sprecher E, Granot M (2011). A psychophysical study of endogenous analgesia: the role of the conditioning pain in the induction and magnitude of conditioned pain modulation. Eur J Pain.

[48] Nir RR, Yarnitsky D, Honigman L, Granot M (2012). Cognitive manipulation targeted at decreasing the conditioning pain perception reduces the efficacy of conditioned pain modulation. Pain.

[49] Perchet C, Frot M, Charmarty A, Flores C, Mazza S, Magnin M, Garcia-Larrea L (2012). Do we activate specifically somatosensory thin fibres with the concentric planar electrode? A scalp and intracranial EEG study. Pain.

[50] Wilson H, Carvalho B, Granot M, Landau R (2013). Temporal stability of conditioned pain modulation in healthy women over four menstrual cycles at the follicular and luteal phases. Pain.

[51] Tousignant-Laflamme Y, Marchand S (2009). Excitatory and inhibitory pain mechanisms during the menstrual cycle in healthy women. Pain.

[52] Rezaii T, Hirschberg AL, Carlstrom K, Ernberg M (2012). The influence of menstrual phases on pain modulation in healthy women. J Pain.

[53] Palit S, Bartley EJ, Kuhn BL, Kerr KL, DelVentura JL, Terry EL, Rhudy JL (2016). Endogenous inhibition of pain and spinal nociception in women with premenstrual dysphoric disorder. J Pain Res.

